# *N*-oleoyl glycine and *N*-oleoyl alanine attenuate alcohol self-administration and preference in mice

**DOI:** 10.1038/s41398-023-02574-4

**Published:** 2023-07-31

**Authors:** Samah Shahen-Zoabi, Reem Smoum, Alexey Bingor, Etty Grad, Alina Nemirovski, Tawfeeq Shekh-Ahmad, Raphael Mechoulam, Rami Yaka

**Affiliations:** Institute for Drug Research (IDR), School of Pharmacy, Faculty of Medicine, The Hebrew University of Jerusalem, Jerusalem, 91120 Israel

**Keywords:** Neuroscience, Molecular neuroscience

## Abstract

The endocannabinoid system (ECS) plays a key modulatory role during synaptic plasticity and homeostatic processes in the brain and has an important role in the neurobiological processes underlying drug addiction. We have previously shown that an elevated ECS response to psychostimulant (cocaine) is involved in regulating the development and expression of cocaine-conditioned reward and sensitization. We therefore hypothesized that drug-induced elevation in endocannabinoids (eCBs) and/or eCB-like molecules (eCB-Ls) may represent a protective mechanism against drug insult, and boosting their levels exogenously may strengthen their neuroprotective effects. Here, we determine the involvement of ECS in alcohol addiction. We first measured the eCBs and eCB-Ls levels in different brain reward system regions following chronic alcohol self-administration using LC–MS. We have found that following chronic intermittent alcohol consumption, *N*-oleoyl glycine (OlGly) levels were significantly elevated in the prefrontal cortex (PFC), and *N*-oleoyl alanine (OlAla) was significantly elevated in the PFC, nucleus accumbens (NAc) and ventral tegmental area (VTA) in a region-specific manner. We next tested whether exogenous administration of OlGly or OlAla would attenuate alcohol consumption and preference. We found that systemic administration of OlGly or OlAla (60 mg/kg, intraperitoneal) during intermittent alcohol consumption significantly reduced alcohol intake and preference without affecting the hedonic state. These findings suggest that the ECS negatively regulates alcohol consumption and boosting selective eCBs exogenously has beneficial effects against alcohol consumption and potentially in preventing relapse.

## Introduction

Alcohol use disorder (AUD) is a chronically relapsing disorder characterized by a progressive escalation from low or moderate to excessive alcohol consumption, and by repeated cycles of intoxication, withdrawal, craving, and relapse [1, 2]. Currently, there are no efficient drugs to treat alcohol addiction and, most importantly, relapse following abstinence. The endocannabinoid system (ECS) is a complex network of receptors and signaling molecules that is found throughout the body and that plays a role in a variety of physiological processes, including pain, mood, appetite, neuroprotection, memory, and reward [3–6]. The ECS includes endocannabinoids (eCBs) and *N*-acyl amino acids [7] (NAAAs) considered eCB-like molecules (eCB-Ls) that are involved in a variety of neurobiological processes underlying drug addiction [8–11]. The presence of the ECS in different brain regions supports a role for endogenous cannabinoid signaling in drug addiction [3]. The anatomical distribution and actions of eCBs are consistent with the behavioral effects of alcohol, including memory disruption, decrease in motor activity, catalepsy, antinociception, and hypothermia [12–16]. eCBs have a strong role in fine-tuning the activity of the mesolimbic dopamine projection to the ventral tegmental area (VTA) and influence nucleus accumbens (NAc) synaptic signaling [11]. Basal levels of eCBs and eCB-Ls differ in specific brain regions and the ECS is upregulated in certain disorders as indicated by an “autoprotective” increased endogenous release of those molecules [17]. Recently it was shown that alcohol administration increases the release of oleoyl ethanolamide (OEA) in the NAc and cerebellum in rats. Systemic administration of OEA significantly attenuated alcohol consumption, blocked cue-induced reinstatement of alcohol-seeking, and reduce the severity of somatic withdrawal symptoms in alcohol-dependent animals [18]. Furthermore, it was shown that OEA counteracts alcohol-induced glial and neuronal alterations in brain regions involved in drug reward [19]. This suggests that OEA may help mitigate the negative effects of alcohol on brain function in relation to reward processing.

Consistent with these studies, it was previously shown that boosting the levels of specific eCB-Ls exogenously can attenuate drug-induced behaviors. Systemic administration of OlGly reduced mecamylamine-precipitated withdrawal responses in nicotine-dependent mice and prevented nicotine condition place preference (CPP) without affecting morphine CPP, demonstrating a degree of selectivity [20]. It was also shown that systemic administration of OlGly interferes with the aversive properties of acute naloxone-precipitated morphine withdrawal in rats [21]. In line with these studies, we have recently demonstrated that administration of OlGly during withdrawal, but not during the acquisition of psychomotor sensitization, attenuated the expression of cocaine sensitization. In addition, the administration of OlGly during the acquisition of cocaine CPP, but not during withdrawal, attenuated the expression of cocaine-conditioned reward [10]. Taken together, these studies strengthen our hypothesis suggesting that boosting the ECS exogenously may have beneficial effects against drug-induced behaviors.

In the current study, we aimed to determine the levels of different eCBs and eCB-Ls following alcohol self-administration using intermittent access to ethanol (EtOH) in a 2-bottle choice procedure (IA2BC). Since there are sex-specific differences in the pattern of alcohol drinking, we examined the levels of eCBs/eCB-Ls in different components of the brain reward system following alcohol self-administration in females and males. Finally, we tested the ability of OlGly and OlAla to attenuate alcohol consumption and preference by systemic administration.

## Materials and methods

### Animals

All the animals used in this study were individually housed under specific pathogen‐free conditions, in standard plastic cages with natural soft sawdust as bedding. 4-week-old female and male C57BL/6 mice (Harlan [Envigo] Laboratories, Jerusalem, Israel) were used. Animals were housed at an ambient temperature of 22–24 °C, humidity at 30–70%, and alternating 12‐h light/dark cycles (lights were on between 7:00 a.m. and 7:00 p.m.) and provided with food and water ad libitum. During the treatment, body weight and food intake were monitored every day. The Institutional Animal Care and Use Committee (IACUC) of the Hebrew University (Jerusalem, Israel) approved all animal care and experimental protocols. Male and female mice were used at age P30–35 for all experiments. Mice were excluded from analysis if weight loss of greater than 10% occurred and randomization carried out where feasible (via Excel). The same investigator analyzed the data.

### Alcohol self-administration—intermittent access to EtOH in a 2-bottle choice procedure (IA2BC)

EtOH and water consumption and preference were measured using a standard 2-bottle choice procedure [22, 23]. Mice were housed individually and received a week of acclimatization and handling. Mice then receive three 24-h sessions of free access to 2-bottle choice (water or 20% EtOH in water) per week, with 24-h and 48-h withdrawal periods during weekdays and weekends, respectively. During the withdrawal periods, mice received two bottles of water and food was available ad libitum. EtOH and water consumption and body weight were measured every 24 h after each session correcting for evaporation and spillage (i.e., the average loss of fluid measured from bottles in 2 empty chambers was subtracted from the amount “consumed”); the left/right position of the bottles was switched to control for any side bias. These data were used to calculate the amount of EtOH consumed and the preference for EtOH relative to water (EtOH consumption/total fluid consumption × 100). OlGly, OlAla, or vehicle was injected by intra peritoneum route (IP, 60 mg/kg) 30 min before each drinking session. Mice that failed to reach a predefined criteria of alcohol intake were excluded to obtain a group of excessive ethanol drinking for both females and males [24].

### Sucrose preference

This procedure was conducted as previously described [25]. Mice had concurrent access to one bottle of sucrose (1% W/V) and one bottle of water for 24 h and the sessions were performed as for EtOH (see above). Solutions intake was normalized to body weight. Sucrose preference was recorded and calculated after 24 h of access.

### Endocannabinoid measurements

Endocannabinoids were extracted and purified as described previously [10]. Reagents and solvents were purchased from Biolab LTD (Jerusalem, Israel) and Sigma-Aldrich (Rehovot, Israel). NMR spectra were recorded at 300 MHz (1H and 13C NMR) using deuterated chloroform (CDCl3, *δ* = 7.26 ppm) with tetramethylsilane (TMS) as the internal standard. Thin-layer chromatography (TLC) was run on silica gel 60F254 plates (Merck). Column chromatography was performed on silica gel 60 Å (Merck). Compounds were located using a UV lamp at 254 nm. OlGly, OlAla, 2-arachidonoyl glycerol (2-AG), arachidonoyl ethanolamine (AEA), oleoyl ethanolamide (OEA), and palmitoyl ethanolamide (PEA) were synthesized as reported [26].

### Liquid chromatography-mass spectrometry analyses

Liquid chromatography-mass spectrometry (LC–MS/MS) analyses were conducted on a Sciex (Framingham, MA, USA) QTRAP® 6500+ mass spectrometer. Air was produced (SF 4 FF compressor, Atlas Copco, Belgium) and purified using an Infinity 1031 nitrogen generator (Peak Scientific, Inchinnan, Scotland). Purified air was used as source and exhaust gases and purified nitrogen as curtain and collision gases. A receiver was placed between the compressor and the nitrogen generator for a large and stable supply of air. The chromatography was performed under reverse phase conditions using a Shimadzu (Kyoto, Japan) UHPLC System, consisting of a Shimadzu CBM-20A communication bus module, Nexera X2 LC-30AD pump, including a Shimadzu DGU-20A5R degasser, a Shimadzu SIL-30AC autosampler, and a Shimadzu CTO-20AC column oven. Liquid chromatographic separation was obtained using 5 μL injections of samples onto a Kinetex 2.6 µm C18 (100*2.1 mm) column from Phenomenex (Torrance, CA, USA). The autosampler was set at 15 °C and the column was maintained at 40°C during the entire analysis.

Data acquisition was performed on a Dell Optiplex XE2 computer using Analyst 1.7.1 and data was analyzed using Sciex OS Software. Gradient elution mobile phases consisted of 0.1% formic acid in water (phase A) and 0.1% formic acid in acetonitrile (phase B). Gradient elution (250 μL/min) is described in Supplementary Table S1. Endocannabinoids were detected in positive and negative ion modes using electron spray ionization (ESI) and multiple reaction monitoring (MRM) mode of acquisition, using d4-PEA as internal standard (IS). The IonDriveTM Turbo V source temperature was set at 550 °C with the ion spray voltage at 4500 V. The curtain gas was set at 55.0 psi. The nebulizer gas (Gas 1) was set to 50 psi, the turbo heater gas (Gas 2) was set to 50 psi. The dwell time was set at 15 msec. The collision energy (CE), declustering potential (DP), and collision cell exit potential (CXP) for the monitored transitions are given in Supplementary Table S2.

### Statistical analysis

Data were analyzed and graphed using GraphPad Prism V.8. All data are presented as mean ± SEM. Unless otherwise noted, group comparisons of one independent variable were tested using an unpaired Student’s *t-test*, and group comparisons of two independent variables were tested using two-way ANOVA or Mixed-effects analysis, with a Sidak *post-hoc* analysis. A probability of *p* < 0.05 was considered statistically significant, indicated by asterisks in the figures. Statistical analysis of Fig. 2 is presented in Supplementary Table 3.

## Results

### Intermittent access to EtOH in a 2-bottle choice procedure in female and male mice

**Fig. 1:**
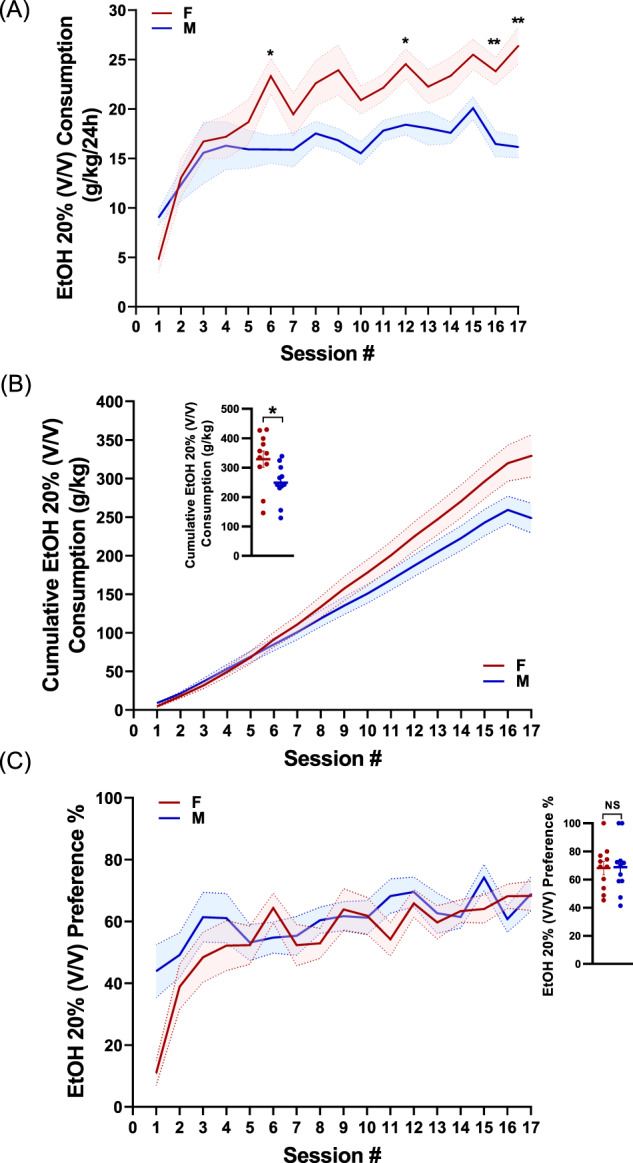
Intermittent access to EtOH induces an escalation of EtOH intake and preference in females and males. **A** Alcohol intake in female (red) and male (blue) mice exposed to intermittent access to 20% EtOH. Female mice exhibited significantly increased EtOH consumption relative to males mice [*N* = 13–15 mice/group; Mixed-effects analysis, followed by Sidak`s multiple comparisons test: *F* (1,27) = 5.509, sex effect *P* = 0.0265; *F* (6.055, 160.1) = 20.6, session *P* < 0.0001; *F* (16, 423) = 3.763, sex × session interaction *P* < 0.0001]. Sidak’s *post-hoc* *P < 0.05, **P < 0.01. **B** Cumulative intake of EtOH (per day) for female or male mice. Inset, absolute EtOH intake for the two groups (*N* = 13–15, *P* = 0.0108, Student’s *t*-test). *P < 0.05. **C** There were no differences in EtOH preference between female and male mice. [*N* = 13-15; Mixed-effects analysis: *F* (1,27) = 0.7908, sex effect *P* = 0.3817; *F* (6.117, 162.5) = 11.98, session *P* < 0.0001; *F* (16, 425) = 2.673, sex × session interaction *P* = 0.0005].

To evaluate the role of the eCBs and eCB-Ls in alcohol use and abuse, we tested the effect of EtOH chronic self-administration on the levels of different eCBs and eCB-Ls, some were shown to have neuroprotective properties [10, 17, 18, 27, 28]. Therefore, female and male mice were tested for EtOH consumption and preference using intermittent access to the EtOH-two-bottle choice (IA2BC) procedure, designed to simulate the repeated episodes of heavy drinking and withdrawal that characterize alcohol abuse [29]. Mice exposed to repeated cycles of free-choice access to 20% EtOH (V/V) and withdrawal during six weeks and their EtOH consumption was measured. Animals reached a high EtOH intake and preference throughout the period of drug exposure (Fig. 1A–C). As was previously demonstrated for both rodents and humans [30–32], female mice exhibited significantly increased EtOH consumption compared to males.

### eCBs and eCB-Ls are differentially altered by voluntary excessive EtOH consumption in the brain reward system

**Fig. 2:**
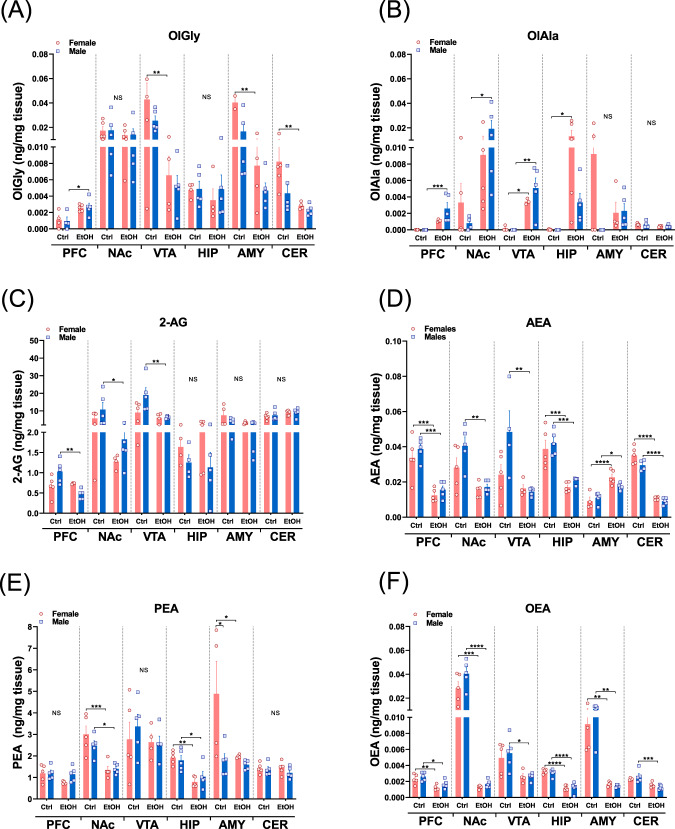
Lipidomic analysis of eCBs and eCB-Ls following chronic EtOH self-administration in female and male mice. **A**–**F** One day following the last EtOH self-administration session, animals were sacrificed and the PFC, NAc, VTA, HIP, AMY, and CER were dissected and analyzed for the expression levels of eCBs using LC–MS. The levels of OlGly, OlAla, 2-AG, AEA, PEA, and OEA are expressed as ng/mg of tissue weight. Two-way ANOVA followed by Sidak’s multiple comparisons revealed that chronic EtOH self-administration significantly elevated OlGly level in the PFC of female mice [red, *N* = 4–5 mice/group; *F* (1,14) = 14.19, *P* = 0.0021; Ctrl vs. EtOH, *P* = 0.0230] and OlAla levels were elevated in PFC and NAc of male (blue) and PFC of female and male mice. *P < 0.05, **P < 0.01, ***P < 0.001. Detailed statistical analysis for all the panels are presented in Supplementary Table S3. NS not significant, PFC prefrontal cortex, NAc nucleus accumbens, VTA ventral tegmental area, HIP hippocampus, AMY amygdala, CER cerebellum, eCBs endocannabinoids, OlGly *N*-oleoyl glycine, OlAla *N*-oleoyl alanine, 2-AG 2-arachidonoyl glycerol, AEA *N*-arachidonoyl ethanolamine, OEA *N*-oleoyl ethanolamide, PEA *N*-palmitoyl ethanolamide.

To characterize changes in the eCB/eCB-L expression profile following chronic alcohol consumption, one day after the last session of the IA2BC, we performed a lipidomic analysis of selected brain regions dissected from female and male mice. The prefrontal cortex (PFC), nucleus accumbens (NAc), ventral tegmental area (VTA), hippocampus (HIP), and amygdala (AMY) which are the principal areas that composed the brain reward system [33], and cerebellum (CER) as a control region, were collected for quantitative profiling of eCBs and eCB-Ls using liquid chromatography-mass spectrometry analysis (LC/MS–MS). The results indicate that the control group compared with EtOH self-administered mice had profoundly different lipid profiles (Fig. 2 and Table 1). We found a general trend of reduction in the expression of OlGly, 2-AG, AEA, PEA, and OEA in all the brain regions tested (Fig. 2A, C–F, and Table 1). However, OlGly in the PFC, OlAla in the PFC, NAc, VTA, and HIP, and AEA in the AMY were elevated compared with the control groups. Moreover, when comparing males and females, we found no difference in the baseline levels of all eCBs/eCB-Ls tested in all brain regions that were analyzed, except for a significant difference in PEA levels found in the AMY (Fig. 2E). Furthermore, we found that the alterations in the expression levels of eCBs/eCB-Ls in the different brain regions were mostly detected in male mice and those found in females mainly, except for OlGly, resembled the changes in male mice (Fig. 2 and Table 1).

**Table 1:** Summary of region-specific eCBs and eCB-Ls alterations following chronic EtOH self-administration.

Brain region\eCB/eCB-L	PFC		NAc		VTA		HIP		AMY		CER	
	F	M	F	M	F	M	F	M	F	M	F	M
OlGly	ns	↑	ns	ns	↓	ns	ns	ns	↓	ns	↓	ns
OlAla	ns	↑	ns	↑	↑	↑	↑	ns	ns	ns	ns	ns
2-AG	ns	↓	ns	↓	ns	↓	ns	ns	ns	ns	ns	ns
AEA	↓	↓	ns	↓	↓	ns	↓	↓	↑	↑	↓	↓
PEA	ns	ns	↓	↓	ns	ns	↓	↓	↓	↓	ns	ns
OEA	↓	↓	↓	↓	ns	↓	↓	↓	↓	↓	ns	↓

Arrows indicate the alteration in the levels of eCBs and eCB-Ls in the various brain regions. Arrows do not represent the exact percent of change (detailed in Fig. 2).

*NS* not significant.

### OlGly and OlAla suppress EtOH self-administration and preference

**Fig. 3:**
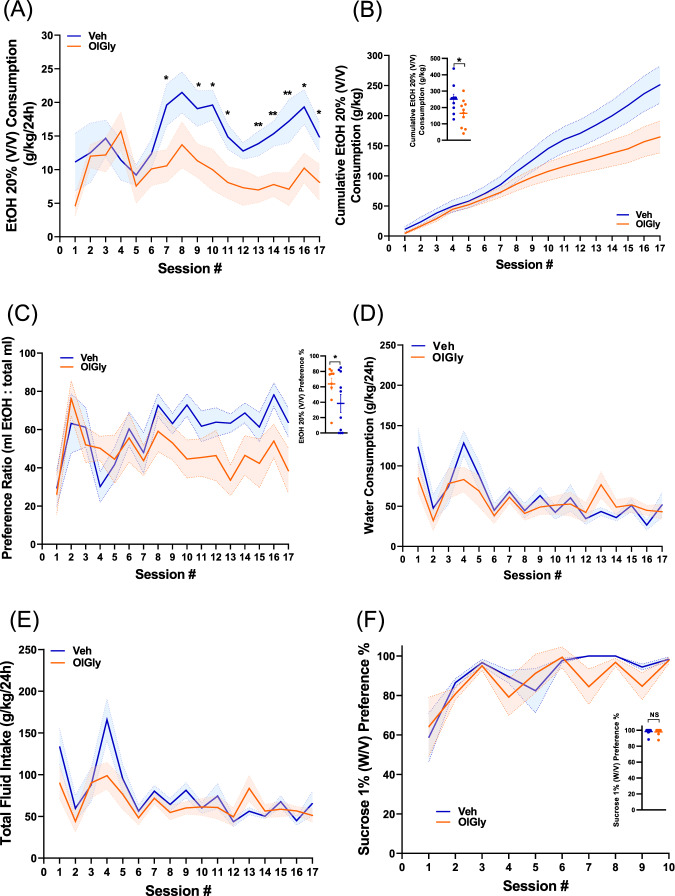
OlGly attenuates EtOH self-administration and preference but not water consumption in male mice. **A** Systemic administration of OlGly (60 mg/kg) before each EtOH self-administration session significantly attenuated EtOH intake [*N* = 9–10; Mixed-effects analysis: *F* (1,17) = 5.349, treatment effect *P* = 0.0335; *F* (5.896, 97.65) = 3.212, session *P* = 0.0067; *F* (16, 265) = 1.791, sex × session interaction *P* = 0.0323]. Student’s *t*-test: *P < 0.05, **P < 0.01. **B** Cumulative EtOH intake (per day) of animals injected with either OlGly (orange, 60 mg/kg) or vehicle (blue). Inset, absolute EtOH intake for the two groups (*N* = 9–10 mice/group, Student’s *t*-test, *P* = 0.0238,). *P < 0.05. **C** OlGly attenuates EtOH preference. Inset, EtOH preference at the last session for the two groups was compared (*N* = 9–10, Student’s *t*-test, *P* = 0.0483), *P < 0.05. **D** Mixed-effects analysis followed by Sidak’s multiple comparisons test revealed no difference in the water consumption between the two groups [*F* (1,9) = 0.1972, *P* = 0.6675]. **E** Mixed-effects analysis, followed by Sidak’s multiple comparisons test revealed no difference in the total fluid intake between the two groups. [*F* (1,9) = 1.322, *P* = 0.2799]. **F** Mixed-effects analysis revealed no significant differences in sucrose (1% W/V) preference between OlGly and vehicle-treated mice [*F* (1,18) = 0.6480, *P* = 0.4313].

We have previously shown that OlGly was elevated in a region-specific manner in the NAc of male mice chronically treated with cocaine, and systemic administration of OlGly (60 mg/kg, IP) negatively regulated the addictive behaviors of cocaine [10]. Considering that in EtOH self-administered mice the levels of OlGly were significantly elevated specifically in the PFC (Fig. 2A), we speculated that the elevation of OlGly may represent a self-defense protective mechanism against alcohol insult. To this end, we tested the ability of OlGly to affect excessive EtOH drinking and preference. Since most of the changes in the expression profile of eCBs/eCB-Ls were found in male mice compared with females, we tested the effect of OlGly in male mice at this stage. Mice were pretreated with either vehicle or OlGly (60 mg/kg, IP) previously shown as the effective dose [10], 30 min before each EtOH consumption day throughout the self-administration sessions. In addition, the total fluid intake (water and EtOH) was measured at the end of each session. As shown in Fig. 3A–C, in OlGly treated animals; EtOH drinking was significantly suppressed as well as EtOH preference. However, the total fluid intake, as well as water consumption, remain unchanged (Fig. 3D, E). Finally, we tested whether OlGly has a non-specific effect on rewards, by testing their preference to natural reward, such as sucrose. To this end, mice were given access to sucrose (1% W/V) in an intermittent access two-bottle choice (water and sucrose) procedure for 4 weeks. No differences in sucrose preference were found between OlGly and vehicle-treated mice (Fig. 3F). These results suggest that OlGly does not affect the animal’s hedonic state.

**Fig. 4:**
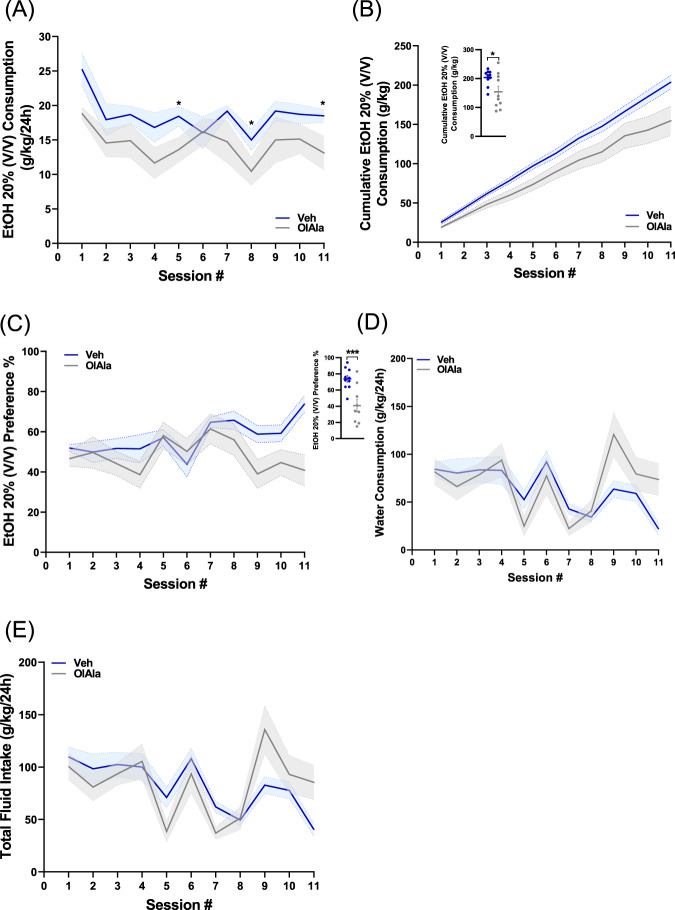
OlAla attenuates EtOH self-administration and preference but not water consumption in male mice. **A** Systemic administration of OlAla (gray, 60 mg/kg) or vehicle (blue) before each EtOH self-administration session significantly attenuated EtOH intake [*N* = 10 mice/group; Mixed-effects analysis: *F* (1,18) = 4.989, treatment effect *P* = 0.0384; *F* (6.118, 108.3) = 4.175, session *P* = 0.0008; *F* (10, 177) = 0.5726, sex × session interaction *P* = 0.8349]. Student’s *t*-test: *P < 0.05. **B** Cumulative EtOH intake (per day) of animals injected with either OlAla or vehicle. Inset, absolute EtOH intake for the two groups (*N* = 10, Student’s *t*-test, *P* = 0.014). *P < 0.05. **C** OlAla attenuates EtOH preference. Inset, EtOH preference was compared at the last session for the two groups (*N* = 10, Student’s *t*-test, *P* = 0.0014). **P < 0.01. **D** Mixed-effects analysis, followed by Sidak’s multiple comparisons test revealed no difference in the water consumption between the two groups. [*F* (1, 9) = 0.3549, *P* = 0.5660]. **E** Mixed-effects analysis, followed by Sidak’s multiple comparisons test revealed no difference in the fluid intake between the two groups. [*F* (1, 9) = 0.02550, *P* = 0.8767].

OlGly is a fatty acid amide susceptible to rapid degradation by fatty acid amide hydrolase (FAAH) [7]. It was previously shown that introducing a methyl group in the α-position of the ethanolamine component of AEA [34] or to the glycine carbon atom in OlGly next to the amide bond (Oleoyl Alanine, OlAla, HU-595) [35] stabilizes the molecule resulting in remarkable metabolic stability and prolonged duration of action [35]. We have previously demonstrated that similar to OlGly, OlAla negatively regulates the behavioral outcomes of cocaine [10]. Since in EtOH self-administered mice the expression of OlAla significantly elevated in the principle areas of the reward system (PFC, NAc, and VTA, Fig. 2B and Table 1), we hypothesized that OlAla will act in a similar manner as OlGly. Therefore, male mice were pretreated with either vehicle or OlAla (60 mg/kg, IP) previously shown as the effective dose [10], 30 min before starting each EtOH session, and their total fluid intake was measured at the end of each session. As shown in Fig. 4A, B, E, OlAla significantly attenuated EtOH intake and preference, without affecting water or total fluid consumption. Taken together, these results suggest that either OlGly or its methylated form OlAla can reduce EtOH consumption and preference.

## Discussion

Our study has directly evaluated EtOH-induced alterations in eCBs and eCBs-like molecules expression both in female and male mice. We found that following intermittent EtOH self-administration there were no significant gender differences in the baseline levels of the eCBs that were tested. However, following EtOH consumption, the levels of eCBs were differentially expressed in a region-specific manner. In addition, we found that the main changes in eCBs were found in both OlGly and OlAla in the PFC and PFC, NAc, and VTA, the three major brain areas that composed the reward system, respectively. Systemic administration of either OlGly or OlAla to male mice significantly reduced EtOH self-administration and preference, without affecting total fluid intake.

As was previously demonstrated, female mice exhibited significantly increased EtOH consumption compared to males [30–32]. In this study, we aimed to evaluate whether this difference is correlated with a different eCBs profile. We first confirmed that female mice consumed more alcohol than male mice using intermittent EtOH self-administration. Indeed, female mice exhibited increased consumption compared with male mice that started to develop following two weeks of EtOH exposure.

The levels of different eCBs and eCB-Ls can markedly fluctuate in different neuropathological conditions. For example, exposure of rats for 21 days to chronic, unpredictable stress in an animal model of depression produced a reduction in the tissue content of AEA in all of the brain regions examined [36, 37]. AEA and 2-AG levels were elevated after traumatic brain injury (TBI) in rat [38] and mouse [39] respectively, and exogenous administration of these compounds following TBI attenuated significantly the brain damage and improve the neurological severity score in these animals. In addition, exogenous administration of OlGly following TBI ameliorates the behavioral alterations associated with TBI in mice, while concomitantly modulating eCB and eCB-like mediator tone [27]. It was shown that the levels of eCB-Ls such as OlGly and OlAla were significantly increased in different regions of the brain following acute inflammation [40].

In addiction studies, reinstatement of cocaine seeking provokes changes in the levels of eCBs (2-AG and AEA) and eCB-Ls (PEA and OEA) in rat brain structures [41], and endogenous fatty acid ethanolamides (PEA and OEA) suppress nicotine-induced activation of mesolimbic dopamine neurons through nuclear receptors [42]. Similarly, it was shown that alcohol administration increases the release of OEA in the NAc and cerebellum in rats, and systemic administration of OEA significantly attenuated alcohol consumption [18].

It was recently demonstrated that boosting the levels of specific eCB-Ls molecules exogenously can attenuate drug-induced behaviors. Systemic administration of OlGly reduced mecamylamine-precipitated withdrawal responses in nicotine-dependent mice and prevented nicotine CPP but not morphine CPP, demonstrating a degree of selectivity [20]. Likewise, systemic administration of OlGly interferes with the aversive properties of acute naloxone-precipitated morphine withdrawal in rats [21]. Correspondingly, we have demonstrated that the administration of OlGly during withdrawal from psychomotor sensitization attenuated the expression of cocaine sensitization. In addition, the administration of OlGly or OlAla during the acquisition of cocaine CPP, decreased the expression of cocaine-conditioned reward [10]. In the current study, we found that within the principal areas of the reward system (PFC, NAc, and VTA), a significant increase of both OlGly and OlAla was measured, except for a decrease of OlGly in the VTA. Based on our previous studies, we, therefore tested the hypothesis that exogenous administration of these compounds will have beneficial behavioral outcomes against drug insult. Since we found no significant differences in the basal levels of all eCBs/eCB-Ls between female and male mice, except for PEA in the amygdala, we tested the effect of OlGly and OlAla in male mice at this stage. Indeed, both lipids reduced EtOH consumption significantly, suggesting that they can play a major role in regulating drug intake. However, since these lipids were administered systemically, their target brain regions and their mechanism of action remained elusive. It was shown that the gut-brain axis can regulate EtOH consumption and preference through peripheral CB1-mediated signaling in ghrelin-producing stomach cells [43]. In addition, it was shown that exogenous administration of OEA reduced alcohol and sucrose consumption and alcohol-seeking behavior which appears to rely on peripheral signaling mechanisms as alcohol self-administration is unaltered by intracerebral PPAR-α agonist administration [18]. Therefore, to determine whether the site of action of OlGly is centrally mediated within the reward system, microinjection of these compounds into the NAc is necessary. Although the precise mechanism of action of OlGly remains unidentified, these findings suggest that the therapeutic potential of eCB-Ls should be further investigated in the context of addiction.

Previous studies have shown that knock-out (K/O) of FAAH resulted in an increase in EtOH consumption and preference [44]. Moreover, in human subjects lower FAAH might alter the positive or negative effects of alcohol and increase urges to drink, thereby contributing to the addiction process [45], suggesting that inhibition of FAAH or its KO positively correlates with increased EtOH consumption [44]. Despite its various described pharmacological properties, the cellular/receptor mechanisms responsible for the actions of OlGly and OlAla are still under debate. OlGly and OlAla may possess neuromodulatory properties as endogenous ligands of PPAR-α and FAAH inhibitors [21, 46]. However, our results demonstrate the ability of these lipids to reduce EtOH consumption and preference. Therefore, the mechanism of action of these eCB-Ls may not involve the inhibition of FAAH. Further studies will be required to clarify the specific mechanisms that mediate their neuroprotective effects.

## Supplementary information

Supplemental Material 1

Supplemental Material 2

Supplemental Material 3

## References

[1] Koob GF (2003). Alcoholism: allostasis and beyond. Alcohol Clin Exp Res.

[2] Koob GF, Volkow ND (2010). Neurocircuitry of addiction. Neuropsychopharmacology.

[3] Cristino L, Bisogno T, Di Marzo V (2020). Cannabinoids and the expanded endocannabinoid system in neurological disorders. Nat Rev Neurol.

[4] Wilson RI, Nicoll RA (2002). Endocannabinoid signaling in the brain. Science.

[5] Mechoulam R, Spatz M, Shohami E (2002). Endocannabinoids and neuroprotection. Sci STKE.

[6] Ligresti A, Petrosino S, Di Marzo V (2009). From endocannabinoid profiling to “endocannabinoid therapeutics.”. Curr Opin Chem Biol.

[7] Bradshaw HB, Rimmerman N, Hu SS, Burstein S, Walker JM. Novel endogenous N-acyl glycines: identification and characterization. Vitamins & Hormones, Vol. 81, Ch. 8:191–205. Elsevier; 2009. 10.1016/S0083-6729(09)81008-X 19647113

[8] Augustin SM, Lovinger DM (2018). Functional relevance of endocannabinoid-dependent synaptic plasticity in the central nervous system. ACS Chem Neurosci.

[9] Spanagel R (2020). Cannabinoids and the endocannabinoid system in reward processing and addiction: from mechanisms to interventions. Dialogues Clin Neurosci.

[10] Shahen-Zoabi S, Smoum R, Beiser T, Nemirovski A, Mechoulam R, Yaka R. N-oleoyl glycine and its derivatives attenuate the acquisition and expression of cocaine-induced behaviors. Cannabis Cannabinoid Res. 2022 10.1089/can.2022.0005. 10.1089/can.2022.0005 35647907

[11] Parsons LH, Hurd YL (2015). Endocannabinoid signalling in reward and addiction. Nat Rev Neurosci.

[12] Ryan C, Butters N (1980). Further evidence for a continuum-of-impairment encompassing male alcoholic Korsakoff patients and chronic alcoholic men. Alcohol Clin Exp Res.

[13] Brandt J, Butters N, Ryan C, Bayog R (1983). Cognitive loss and recovery in long-term alcohol abusers. Arch Gen Psychiatry.

[14] Herkenham M, Lynn AB, Johnson MR, Melvin LS, de Costa BR, Rice KC (1991). Characterization and localization of cannabinoid receptors in rat brain: a quantitative in vitro autoradiographic study. J Neurosci.

[15] Compton DR, Rice KC, De Costa BR, Razdan RK, Martin BR (1993). Cannabinoid structure-activity relationships: Correlation of receptor binding and in vivo activities. J Pharmacol Exp Ther.

[16] Fadda F, Rossetti ZL (1998). Chronic ethanol consumption: from neuroadaptation to neurodegeneration. Prog Neurobiol.

[17] Pertwee RG (2014). Elevating endocannabinoid levels: pharmacological strategies and potential therapeutic applications. Proc Nutr Soc.

[18] Bilbao A, Serrano A, Cippitelli A, Pavón FJ, Giuffrida A, Suárez J (2016). Role of the satiety factor oleoylethanolamide in alcoholism. Addict Biol.

[19] Rivera P, Silva-Peña D, Blanco E, Vargas A, Arrabal S, Serrano A (2019). Oleoylethanolamide restores alcohol-induced inhibition of neuronal proliferation and microglial activity in striatum. Neuropharmacology.

[20] Donvito G, Piscitelli F, Muldoon P, Jackson A, Vitale RM, D’Aniello E (2019). N-Oleoyl-glycine reduces nicotine reward and withdrawal in mice. Neuropharmacology.

[21] Petrie GN, Wills KL, Piscitelli F, Smoum R, Limebeer CL, Rock EM (2019). Oleoyl glycine : interference with the aversive effects of acute naloxone-precipitated MWD, but not morphine reward, in male Sprague – Dawley rats. Psychopharmacology.

[22] Stuber GD, Hopf FW, Hahn J, Cho SL, Guillory A, Bonci A (2008). Voluntary ethanol intake enhances excitatory synaptic strength in the ventral tegmental area. Alcohol Clin Exp Res.

[23] Simms JA, Steensland P, Medina B, Abernathy KE, Chandler LJ, Wise R (2008). Intermittent access to 20% ethanol induces high ethanol consumption in Long-Evans and Wistar rats. Alcohol Clin Exp Res.

[24] Carnicella S, Kharazia V, Jeanblanc J, Janak PH, Ron D (2008). GDNF is a fast-acting potent inhibitor of alcohol consumption and relapse. Proc Natl Acad Sci USA.

[25] Warnault V, Darcq E, Levine A, Barak S, Ron D (2013). Chromatin remodeling–a novel strategy to control excessive alcohol drinking. Transl Psychiatry.

[26] Sheskin T, Hanus L, Slager J, Vogel Z, Mechoulam R (1997). Structural requirements for binding of anandamide-type compounds to the brain cannabinoid receptor. J Med Chem.

[27] Piscitelli F, Guida F, Luongo L, Iannotti FA, Boccella S, Verde R (2020). Protective effects of N-oleoylglycine in a mouse model of mild traumatic brain injury. ACS Chem Neurosci.

[28] Lauritano A, Cipollone I, Verde R, Kalkan H, Moriello C, Iannotti FA (2022). The endocannabinoidome mediator N-oleoylglycine is a novel protective agent against 1-methyl-4-phenyl-pyridinium-induced neurotoxicity. Front Aging Neurosci.

[29] Wise RA (1973). Voluntary ethanol intake in rats following exposure to ethanol on various schedules. Psychopharmacologia.

[30] Middaugh LD, Kelley BM, Bandy AL, McGroarty KK (1999). Ethanol consumption by C57BL/6 mice: influence of gender and procedural variables. Alcohol.

[31] Anker JJ, Carroll ME Females Are More Vulnerable to Drug Abuse than Males: Evidence from Preclinical Studies and the Role of Ovarian Hormones. In: Neill JC, Kulkarni J, editors. Biological basis of sex differences in psychopharmacology. Berlin, Heidelberg: Springer Berlin Heidelberg; 2011. p. 73–96. 10.1007/7854_2010_93 21769724

[32] Ziv Y, Rahamim N, Lezmy N, Even-Chen O, Shaham O, Malishkevich A (2019). Activity-dependent neuroprotective protein (ADNP) is an alcohol-responsive gene and negative regulator of alcohol consumption in female mice. Neuropsychopharmacology.

[33] Cooper S, Robison AJ, Mazei-Robison MS (2017). Reward circuitry in addiction. Neurotherapeutics.

[34] Abadji V, Lin S, Taha G, Griffin G, Stevenson LA, Pertwee RG (1994). (R)-methanandamide: a chiral novel anandamide possessing higher potency and metabolic stability. J Med Chem.

[35] Ayoub SM, Smoum R, Farag M, Atwal H, Collins SA, Rock EM (2020). Oleoyl alanine (HU595): a stable monomethylated oleoyl glycine interferes with acute naloxone precipitated morphine withdrawal in male rats. Psychopharmacology.

[36] González S, Valenti M, de Miguel R, Fezza F, Fernández-Ruiz J, Di Marzo V (2004). Changes in endocannabinoid contents in reward-related brain regions of alcohol-exposed rats, and their possible relevance to alcohol relapse. Br J Pharmacol.

[37] Hill MN, Carrier EJ, McLaughlin RJ, Morrish AC, Meier SE, Hillard CJ (2008). Regional alterations in the endocannabinoid system in an animal model of depression: effects of concurrent antidepressant treatment. J Neurochem.

[38] Hansen HH, Schmid PC, Bittigau P, Lastres-Becker I, Berrendero F, Manzanares J (2001). Anandamide, but not 2-arachidonoylglycerol, accumulates during in vivo neurodegeneration. J Neurochem.

[39] Shohami E, Cohen-Yeshurun A, Magid L, Algali M, Mechoulam R (2011). Endocannabinoids and traumatic brain injury. Br J Pharmacol.

[40] Raboune S, Stuart JM, Leishman E, Takacs SM, Rhodes B, Basnet A (2014). Novel endogenous N-acyl amides activate TRPV1-4 receptors, BV-2 microglia, and are regulated in brain in an acute model of inflammation. Front Cell Neurosci.

[41] Bystrowska B, Frankowska M, Smaga I, Niedzielska-Andres E, Pomierny-Chamioło L, Filip M (2019). Cocaine-induced reinstatement of cocaine seeking provokes changes in the endocannabinoid and N-acylethanolamine levels in rat brain structures. Molecules.

[42] Melis M, Pillolla G, Luchicchi A, Muntoni AL, Yasar S, Goldberg SR (2008). Endogenous fatty acid ethanolamides suppress nicotine-induced activation of mesolimbic dopamine neurons through nuclear receptors. J Neurosci.

[43] Godlewski G, Cinar R, Coffey NJ, Liu J, Jourdan T, Mukhopadhyay B (2019). Targeting peripheral CB1 receptors reduces ethanol intake via a gut-brain axis. Cell Metab.

[44] Blednov YA, Cravatt BF, Boehm SL, Walker D, Harris RA (2007). Role of endocannabinoids in alcohol consumption and intoxication: studies of mice lacking fatty acid amide hydrolase. Neuropsychopharmacology.

[45] Best LM, Hendershot CS, Buckman JF, Jagasar S, McPhee MD, Muzumdar N, et al. Association between fatty acid amide hydrolase and alcohol response phenotypes: a positron emission tomography imaging study with [11C]CURB in heavy-drinking youth. Biol Psychiatry. 2022 (in the press). 10.1016/j.biopsych.2022.11.022. 10.1016/j.biopsych.2022.11.022 36868890

[46] Iannotti FA, Vitale RM (2021). The endocannabinoid system and PPARs: focus on their signalling crosstalk, action and transcriptional regulation. Cells.

