# Supplementary material for: N-oleoyl glycine and N-oleoyl alanine attenuate alcohol self-administration and preference in mice
Source: Transl Psychiatry. 2023 Jul 31;13:273. doi: 10.1038/s41398-023-02574-4 (PMC10390512; doi:10.1038/s41398-023-02574-4)
Supplement: Supplementary file 1 — Supplemental Material 1 [file 41398_2023_2574_MOESM1_ESM.pdf]

Supplementary Table S1: Gradient elution mobile phases consisted of 0.1% formic acid in water (phase A) and 0.1% formic acid in acetonitrile (phase B).

Time	Phase A, %	Phase B, %
3.0	50	50
10.0	30	70
29.5	27	73
30.0	10	90
35.0	10	90
35.5	50	50
40	50	50
