# Supplementary material for: N-oleoyl glycine and N-oleoyl alanine attenuate alcohol self-administration and preference in mice
Source: Transl Psychiatry. 2023 Jul 31;13:273. doi: 10.1038/s41398-023-02574-4 (PMC10390512; doi:10.1038/s41398-023-02574-4)
Supplement: Supplementary file 2 — Supplemental Material 2 [file 41398_2023_2574_MOESM2_ESM.pdf]

Supplementary Table S2A: Monitored MRM transitions in positive mode.

Compounds	Molecular ion $[M+H]^+$ (m/z)	Fragment (m/z)	DP (volts)	CE (volts)	CXP (volts)
2AG	379.2	287.1 (quantifier)	70	19	14
		91 (qualifier)	70	67	10
AEA	348.2	287.1 (quantifier)	60	13	16
		62 (qualifier)	60	13	8
PEA	300.3	283.2 (quantifier)	150	19	24
		62 (qualifier)	150	17	8
OEA	326.3	61.9 (quantifier)	146	21	24
		309.1 (qualifier)	146	21	42
OlAla	354.4	90 (quantifier)	60	17	10
		265.2 (quantifier)	60	17	14
d_4-PEA	304.2	286.9 (quantifier)	60	21	24
		62 (qualifier)	60	19	28

Supplementary Table S2B: Monitored MRM transitions in negative mode.

Compounds	Molecular ion $[M-H]^-$ (m/z)	Fragment (m/z)	DP (volts)	CE (volts)	CXP (volts)
OlGly	338.3	74 (quantifier)	-50	-24	-11
		294.2 (qualifier)	-50	-26	-15
