# Supplementary material for: N-oleoyl glycine and N-oleoyl alanine attenuate alcohol self-administration and preference in mice
Source: Transl Psychiatry. 2023 Jul 31;13:273. doi: 10.1038/s41398-023-02574-4 (PMC10390512; doi:10.1038/s41398-023-02574-4)
Supplement: Supplementary file 3 — Supplemental Material 3 [file 41398_2023_2574_MOESM3_ESM.pdf]

Supplementary Table S3: Detailed statistical analysis for all panels in figure 2

Figure	Test		*F* value	DF	*P*-value
**2A (PFC)**	2-Way ANOVA followed by Sidak's post-hoc	Interaction	0.1153	1, 14	0.7393
		Treatment	14.19	1, 14	0.0021
		Sex	0.005018	1, 14	0.9445
**2A (NAc)**	2-Way ANOVA followed by Sidak's post-hoc	Interaction	0.0003681	1, 16	0.9849
		Treatment	0.7293	1, 16	0.4057
		Sex	0.009176	1, 16	0.9249
**2A (VTA)**	2-Way ANOVA followed by Sidak's post-hoc	Interaction	1.122	1, 15	0.3063
		Treatment	13.88	1, 15	0.0020
		Sex	1.518	1, 15	0.2369
**2A (HIP)**	2-Way ANOVA followed by Sidak's post-hoc	Interaction	0.22442	1, 14	0.6432
		Treatment	0.2453	1, 14	0.6281
		Sex	0.3232	1, 14	0.5787
**2A (AMY)**	2-Way ANOVA followed by Sidak's post-hoc	Interaction	4.681	1, 12	0.0514
		Treatment	21.86	1, 12	0.0005
		Sex	7.841	1, 12	0.0160
**2A (CER)**	2-Way ANOVA followed by Sidak's post-hoc	Interaction	2.255	1, 15	0.1540
		Treatment	11.12	1, 15	0.0045
		Sex	3.872	1, 15	0.0679
**2B (PFC)**	2-Way ANOVA followed by Sidak's post-hoc	Interaction	3.628	1, 15	0.0762
		Treatment	22.70	1, 15	0.0003
		Sex	3.628	1, 15	0.0762
**2B (NAc)**	2-Way ANOVA followed by Sidak's post-hoc	Interaction	1.964	1, 15	0.1814
		Treatment	7.213	1, 15	0.0169
		Sex	0.7077	1, 15	0.4134
**2B (VTA)**	2-Way ANOVA followed by Sidak's post-hoc	Interaction	1.179	1, 12	0.2989
		Treatment	26.12	1, 12	0.0003
		Sex	0.8393	1, 12	0.3777
**2B (HIP)**	2-Way ANOVA followed by Sidak's post-hoc	Interaction	2.827	1, 14	0.1149
		Treatment	8.053	1, 14	0.0132
		Sex	2.861	1, 14	0.1129
**2B (AMY)**	2-Way ANOVA followed by Sidak's post-hoc	Interaction	2.818	1, 13	0.1171
		Treatment	0.7417	1, 13	0.4047
		Sex	2.557	1, 13	0.1338
**2B (CER)**	2-Way ANOVA followed by Sidak's post-hoc	Interaction	0.006330	1, 15	0.9376
		Treatment	4.774	1, 15	0.0452
		Sex	0.009555	1, 15	0.9234
**2C (PFC)**	2-Way ANOVA followed by Sidak's post-hoc	Interaction	11.04	1, 15	0.0046
		Treatment	6.959	1, 15	0.0186
		Sex	0.2458	1, 15	0.6272
**2C (NAc)**	2-Way ANOVA followed by Sidak's post-hoc	Interaction	0.8880	1, 15	0.3610
		Treatment	7.893	1, 15	0.0132
		Sex	1.375	1, 15	0.2593

**2C (VTA)**	2-Way ANOVA followed by Sidak's post-hoc	Interaction	3.149	1, 15	0.0963
		Treatment	8.450	1, 15	0.0108
		Sex	3.500	1, 15	0.0810
**2C (HIP)**	2-Way ANOVA followed by Sidak's post-hoc	Interaction	0.8661	1, 14	0.3678
		Treatment	0.4501	1, 14	0.5132
		Sex	3.220	1, 14	0.0943
**2C (AMY)**	2-Way ANOVA followed by Sidak's post-hoc	Interaction	1.153	1, 15	0.2999
		Treatment	5.213	1, 15	0.0374
		Sex	2.276	1, 15	0.1522
**2C (CER)**	2-Way ANOVA followed by Sidak's post-hoc	Interaction	0.06768	1, 16	0.7981
		Treatment	3.522	1, 16	0.0789
		Sex	0.3251	1, 16	0.5765
**2D (PFC)**	2-Way ANOVA followed by Sidak's post-hoc	Interaction	0.07199	1, 16	0.7919
		Treatment	47.83	1, 16	<0.0001
		Sex	1.615	1, 16	0.2220
**2D (NAc)**	2-Way ANOVA followed by Sidak's post-hoc	Interaction	1.740	1, 16	0.2057
		Treatment	21.06	1, 16	0.0003
		Sex	3.199	1, 16	0.0926
**2D (VTA)**	2-Way ANOVA followed by Sidak's post-hoc	Interaction	4.288	1, 14	0.0573
		Treatment	11.01	1, 14	0.0051
		Sex	3.187	1, 14	0.0959
**2D (HIP)**	2-Way ANOVA followed by Sidak's post-hoc	Interaction	0.002092	1, 15	0.9641
		Treatment	44.39	1, 15	<0.0001
		Sex	1.217	1, 15	0.2873
**2D (AMY)**	2-Way ANOVA followed by Sidak's post-hoc	Interaction	5.145	1, 15	0.0385
		Treatment	37.06	1, 15	<0.0001
		Sex	1.199	1, 15	0.2907
**2D (CER)**	2-Way ANOVA followed by Sidak's post-hoc	Interaction	2.099	1, 15	0.1680
		Treatment	259.7	1, 15	<0.0001
		Sex	6.863	1, 15	0.0193
**2E (PFC)**	2-Way ANOVA followed by Sidak's post-hoc	Interaction	1.593	1, 15	0.2262
		Treatment	2.969	1, 15	0.1054
		Sex	2.013	1, 15	0.1764
**2E (NAc)**	2-Way ANOVA followed by Sidak's post-hoc	Interaction	1.395	1, 16	0.2548
		Treatment	31.66	1, 16	<0.0001
		Sex	0.9242	1, 16	0.3507
**2E (VTA)**	2-Way ANOVA followed by Sidak's post-hoc	Interaction	0.3327	1, 15	0.5726
		Treatment	0.6512	1, 15	0.4323
		Sex	0.2263	1, 15	0.6411
**2E (HIP)**	2-Way ANOVA followed by Sidak's post-hoc	Interaction	0.9649	1, 16	0.3406
		Treatment	25.30	1, 16	0.0001
		Sex	0.09922	1, 16	0.7568
**2E (AMY)**	2-Way ANOVA followed by Sidak's post-hoc	Interaction	3.894	1, 14	0.0685
		Treatment	5.025	1, 14	0.0417
		Sex	6.379	1, 14	0.0242
**2E (CER)**	2-Way ANOVA followed by Sidak's post-hoc	Interaction	0.5163	1, 16	0.4828
		Treatment	0.3337	1, 16	0.5715
		Sex	1.169	1, 16	0.2956

**2F (PFC)**	2-Way ANOVA followed by Sidak's post-hoc	Interaction	0.07125	1, 16	0.7929
		Treatment	19.77	1, 16	0.0004
		Sex	2.708	1, 16	0.1193
**2F (NAc)**	2-Way ANOVA followed by Sidak's post-hoc	Interaction	2.492	1, 16	0.1340
		Treatment	76.75	1, 16	<0.0001
		Sex	2.854	1, 16	0.1105
**2F (VTA)**	2-Way ANOVA followed by Sidak's post-hoc	Interaction	0.1837	1, 15	0.6743
		Treatment	13.99	1, 15	0.0020
		Sex	0.1754	1, 15	0.6813
**2F (HIP)**	2-Way ANOVA followed by Sidak's post-hoc	Interaction	0.2584	1, 16	0.6181
		Treatment	95.15	1, 16	<0.0001
		Sex	0.2088	1, 16	0.6539
**2F (AMY)**	2-Way ANOVA followed by Sidak's post-hoc	Interaction	0.7895	1, 15	0.3883
		Treatment	50.15	1, 15	<0.0001
		Sex	0.4695	1, 15	0.5037
**2F (CER)**	2-Way ANOVA followed by Sidak's post-hoc	Interaction	2.467	1, 15	0.1371
		Treatment	23.55	1, 15	0.0002
		Sex	0.01076	1, 15	0.9188
**2G (PFC)**	2-Way ANOVA followed by Sidak's post-hoc	Interaction	0.6183	1, 16	0.4432
		Treatment	106.0	1, 16	<0.0001
		Sex	0.6183	1, 16	0.4432
**2G (NAc)**	2-Way ANOVA followed by Sidak's post-hoc	Interaction	3.586	1, 15	0.0777
		Treatment	0.2764	1, 15	0.6067
		Sex	0.1133	1, 15	0.7411
**2G (VTA)**	2-Way ANOVA followed by Sidak's post-hoc	Interaction	1.029	1, 15	0.3265
		Treatment	4.459	1, 15	0.0519
		Sex	0.9646	1, 15	0.3416
**2G (HIP)**	2-Way ANOVA followed by Sidak's post-hoc	Interaction	0.07412	1, 16	0.7889
		Treatment	1.055	1, 16	0.3196
		Sex	3.822	1, 16	0.0683
**2G (AMY)**	2-Way ANOVA followed by Sidak's post-hoc	Interaction	0.1044	1, 12	0.7521
		Treatment	0.2470	1, 12	0.6282
		Sex	0.7580	1, 12	0.4010
**2G (CER)**	2-Way ANOVA followed by Sidak's post-hoc	Interaction	4.746	1, 14	0.0469
		Treatment	1.145	1, 14	0.3027
		Sex	6.565	1, 14	0.0226

Multiple comparisons:

FIG. 2A (OlGly)

Sidak`s multiple comparisons test	Summary	Adjusted P Value
**PFC**		
Females Ctrl vs. Females EtOH	ns	0.0581
Males Ctrl vs. Males EtOH	*	0.0230
**NAc**		
Females Ctrl vs. Females EtOH	ns	0.7936

Males Ctrl vs. Males EtOH	ns	0.8092
**VTA**		
Females Ctrl vs. Females EtOH	**	0.0099
Males Ctrl vs. Males EtOH	ns	0.1368
**HIP**		
Females Ctrl vs. Females EtOH	ns	0.7756
Males Ctrl vs. Males EtOH	ns	0.9998
**AMY**		
Females Ctrl vs. Females EtOH	**	0.0023
Males Ctrl vs. Males EtOH	ns	0.1063
**CER**		
Females Ctrl vs. Females EtOH	**	0.0092
Males Ctrl vs. Males EtOH	ns	0.3624

FIG. 2B (OlAla)

Sidak`s multiple comparisons test	Summary	Adjusted P Value
**PFC**		
Females Ctrl vs. Females EtOH	ns	0.1317
Males Ctrl vs. Males EtOH	***	0.0004
**NAc**		
Females Ctrl vs. Females EtOH	ns	0.5955
Males Ctrl vs. Males EtOH	*	0.0263
**VTA**		
Females Ctrl vs. Females EtOH	*	0.0398
Males Ctrl vs. Males EtOH	**	0.0010
**HIP**		
Females Ctrl vs. Females EtOH	*	0.0129
Males Ctrl vs. Males EtOH	ns	0.6719
**AMY**		
Females Ctrl vs. Females EtOH	ns	0.1964
Males Ctrl vs. Males EtOH	ns	0.8088
**CER**		
Females Ctrl vs. Females EtOH	ns	0.2613
Males Ctrl vs. Males EtOH	ns	0.2702

FIG. 2C (2-AG)

Sidak`s multiple comparisons test	Summary	Adjusted P Value
**PFC**		
Females Ctrl vs. Females EtOH	ns	0.8738

Males Ctrl vs. Males EtOH	**	0.0012
**NAc**		
Females Ctrl vs. Females EtOH	ns	0.3900
Males Ctrl vs. Males EtOH	*	0.0305
**VTA**		
Females Ctrl vs. Females EtOH	ns	0.6959
Males Ctrl vs. Males EtOH	**	0.0077
**HIP**		
Females Ctrl vs. Females EtOH	ns	0.4765
Males Ctrl vs. Males EtOH	ns	0.9795
**AMY**		
Females Ctrl vs. Females EtOH	ns	0.0702
Males Ctrl vs. Males EtOH	ns	0.6303
**CER**		
Females Ctrl vs. Females EtOH	ns	0.2780
Males Ctrl vs. Males EtOH	ns	0.4668

FIG. 2D (AEA)

Sidak`s multiple comparisons test	Summary	Adjusted P Value
**PFC**		
Females Ctrl vs. Females EtOH	***	0.0005
Males Ctrl vs. Males EtOH	***	0.0002
**NAc**		
Females Ctrl vs. Females EtOH	ns	0.0677
Males Ctrl vs. Males EtOH	**	0.0014
**VTA**		
Females Ctrl vs. Females EtOH	ns	0.6308
Males Ctrl vs. Males EtOH	**	0.0038
**HIP**		
Females Ctrl vs. Females EtOH	***	0.0004
Males Ctrl vs. Males EtOH	***	0.0008
**AMY**		
Females Ctrl vs. Females EtOH	****	<0.0001
Males Ctrl vs. Males EtOH	*	0.0276
**CER**		
Females Ctrl vs. Females EtOH	****	<0.0001
Males Ctrl vs. Males EtOH	****	<0.0001

FIG. 2E (PEA)

Sidak`s multiple comparisons test	Summary	Adjusted P Value

PFC

Females Ctrl vs. Females EtOH	ns	0.1129
Males Ctrl vs. Males EtOH	ns	0.9332

NAc

Females Ctrl vs. Females EtOH	***	0.0004
Males Ctrl vs. Males EtOH	*	0.0125

VTA

Females Ctrl vs. Females EtOH	ns	0.9847
Males Ctrl vs. Males EtOH	ns	0.5500

HIP

Females Ctrl vs. Females EtOH	**	0.0012
Males Ctrl vs. Males EtOH	*	0.0224

AMY

Females Ctrl vs. Females EtOH	*	0.0267
Males Ctrl vs. Males EtOH	ns	0.9755

CER

Females Ctrl vs. Females EtOH	ns	0.9939
Males Ctrl vs. Males EtOH	ns	0.6069

FIG. 2F (OEA)

Sidak`s multiple comparisons test	Summary	Adjusted P Value
**PFC**		
Females Ctrl vs. Females EtOH	**	0.0084
Males Ctrl vs. Males EtOH	*	0.0185
**NAc**		
Females Ctrl vs. Females EtOH	***	0.0002
Males Ctrl vs. Males EtOH	****	<0.0001
**VTA**		
Females Ctrl vs. Females EtOH	ns	0.0745
Males Ctrl vs. Males EtOH	*	0.0165
**HIP**		
Females Ctrl vs. Females EtOH	****	<0.0001
Males Ctrl vs. Males EtOH	****	<0.0001
**AMY**		
Females Ctrl vs. Females EtOH	**	0.0014
Males Ctrl vs. Males EtOH	****	<0.0001
**CER**		
Females Ctrl vs. Females EtOH	ns	0.0773
Males Ctrl vs. Males EtOH	***	0.0006
